# A Single Valine Residue Plays an Essential Role in Peripherin/rds Targeting to Photoreceptor Outer Segments

**DOI:** 10.1371/journal.pone.0054292

**Published:** 2013-01-14

**Authors:** Raquel Y. Salinas, Sheila A. Baker, Sidney M. Gospe, Vadim Y. Arshavsky

**Affiliations:** 1 Department of Pharmacology and Cancer Biology, Duke University Medical Center, Durham, North Carolina, United States of America; 2 Department of Biochemistry, University of Iowa, Iowa City, Iowa, United States of America; 3 Department of Ophthalmology, Duke University Medical Center, Durham, North Carolina, United States of America; Dalhousie University, Canada

## Abstract

Peripherin/retinal degeneration slow (rds) is an integral membrane protein specifically localized to the light-sensing organelle of the photoreceptor cell, the outer segment. Within the outer segment, peripherin is found at the edges of photoreceptor discs, where it plays a critical role in disc morphogenesis and maintenance. Peripherin loss or mutations are often associated with severe forms of visual impairments. Like all other resident outer segment proteins, peripherin is synthesized in the photoreceptor cell body and subsequently transported to the outer segment. In an effort to further examine peripherin’s delivery to outer segments, we undertook a careful examination of its targeting sequence. Using a fluorescently labeled reporter expressed in the rods of transgenic tadpoles, we narrowed peripherin’s targeting sequence to ten amino acids within its C-terminal tail. This small stretch of amino acid residues is both necessary and sufficient for outer segment targeting. We also conducted alanine scanning of all residues within this sequence and found that only a single residue, valine at position 332, is essential for outer segment targeting. This valine is conserved in all species and its mutation is sufficient to completely abrogate the targeting of full-length peripherin in mouse rods.

## Introduction

Vision is initiated in the retina where light is captured by the outer segment organelle of photoreceptor cells. The outer segment is a modified primary cilium that contains large quantities of proteins involved in visual signal transduction. Similar to all cilia, the outer segment lacks the machinery required to synthesize proteins and therefore relies on the import of proteins produced in the cell body of photoreceptor cells. The importance of accurate protein targeting to the outer segment is highlighted by observations that defects in protein targeting result in retinal degenerative diseases [1]–3.

Membrane proteins destined for the outer segment are synthesized in the endoplasmic reticulum, transported through the Golgi, and then sorted at the trans-Golgi network into transport vesicles specifically directed to the outer segment. The fidelity of sorting is guided by targeting signals, which are short stretches of amino acid residues encoding protein localization information [4], [5]. These targeting signals often reside within a protein’s cytoplasmic domain and are deciphered by protein sorting complexes present at the trans-Golgi. Only two targeting signals responsible for directing membrane proteins to the outer segment have been reported thus far. One signal is VXPX, which is shared by rhodopsin, cone opsins, and the photoreceptor-specific retinol dehydrogenase [6]–[9], as well as several other proteins targeted to primary and sensory cilia in other cell types [10]–[12]. In photoreceptors, this signal interacts with a small GTPase Arf4, which defines rhodopsin packaging into transport vesicles for outer segment delivery [13], [14].

The second known targeting sequence resides within the C-terminus of peripherin/retinal degeneration slow (also known as rds or peripherin-2, hereafter referred to as peripherin) [15]. Peripherin is a member of the tetraspanin family with the characteristic topology of four transmembrane domains, a large extracellular/intradiscal loop, and relatively short cytoplasmic N and C-termini. Peripherin localizes specifically to the rims of outer segment disc membranes and plays a crucial role in outer segment morphogenesis [16], [17]. This role is particularly highlighted in *rds* mice, in which the peripherin gene is severely truncated, essentially making them a peripherin knockout [18]. These mice completely lack photoreceptor outer segments and instead display rudimentary stumps lacking disc structures [19], [20]. Consistent with mouse studies showing a requirement for peripherin in outer segment morphogenesis, over 90 different mutations in human peripherin have been associated with visual impairments (http://www.retina-international.org/files/sci-news/rdsmut.htm).

Unlike rhodopsin, it is unclear how peripherin is delivered to the outer segment. One of the first studies to examine this question showed that peripherin accumulates in intracellular vesicles while rhodopsin accumulates in the plasma membrane of photoreceptors in detached cat retinas [21]. Results obtained in dying photoreceptors are difficult to interpret, but this finding may be viewed as indirect evidence that under normal conditions peripherin and rhodopsin utilize separate transport pathways. No mislocalized peripherin was found in any mouse models in which rhodopsin is knocked out or mislocalized [22], [23], thus establishing that peripherin can be delivered independently of rhodopsin. However, this does not preclude peripherin from travelling in the same vesicles as rhodopsin under normal conditions. Studies examining photoreceptor targeting of C-terminal fragments of peripherin fused to a GFP reporter construct revealed that an amino acid stretch (residues 317–336) is necessary to target a reporter to *Xenopus* rod outer segments [15]. Notably, this twenty amino acid sequence overlaps with a functional domain of peripherin implicated in membrane fusion [24]–[27].

The essential requirement for peripherin in outer segment morphogenesis prompted us to further characterize its outer segment targeting. Targeting signals are often 4–7 amino acids long, with only 2–3 residues being critical to the specific targeting of the protein [28], [29]. Our goals were to narrow the previously identified peripherin targeting sequence, determine whether the targeting and the fusogenic domains were separable, and identify its most critical residues. Here we show that the targeting sequence is confined within ten amino acid residues, which do not overlap with the reported fusogenic domain, and that only a single amino acid within this region is irreplaceable – a highly conserved valine at position 332.

## Results and Discussion

### Peripherin C-terminus Contains a Ten Amino Acid Targeting Sequence

**Figure 1:**
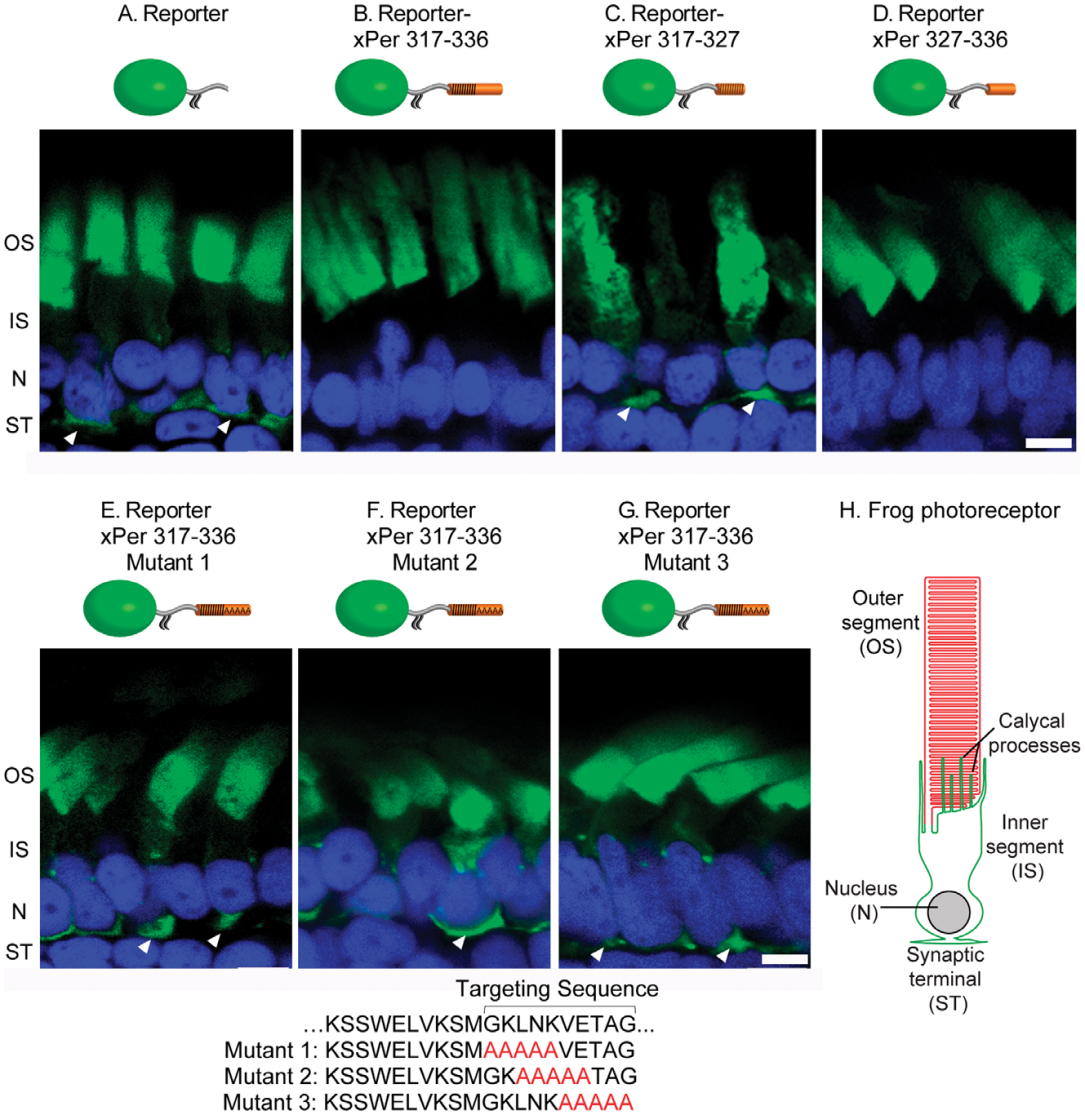
The peripherin targeting signal is contained within a ten amino acid residue stretch. Panels show confocal images of transgenic frog retinas expressing the reporter construct YFP-xRhoCTΔ5 (green) fused to the fragments of the peripherin C-terminus illustrated in cartoons above the corresponding panels. Partial mislocalization of several constructs from rod outer segments is marked by white arrowheads. (A) The YFP-xRhoCTΔ5 reporter. (B) The reporter fused to xPer 317–336. (C) The reporter fused to xPer 317–327. (D) The reporter fused to xPer 327–336. (E–G) The reporter fused to xPer 317–336 sequences containing polyalanine amino acid substitutions indicated below the panels. Abbreviations are: OS – outer segment, IS – inner segment, N – nuclei, ST – synaptic termini. The nuclei (blue) are stained with Hoechst; scale bar: 5 µm. (H) A schematic of a frog rod photoreceptor illustrating its principle compartments.

In an experimental approach modeled after Tam and colleagues [7], [15], we used a reporter construct consisting of YFP fused to the *Xenopus* rhodopsin C‐terminus lacking the VXPX targeting motif (YFP‐xRhoCTΔ5). This construct retains two palmitoylated cysteine residues critical for membrane attachment, but lacks specific outer segment targeting information. When expressed in *Xenopus* under the rhodopsin promoter, the majority of the reporter localizes to the outer segment, but a distinct portion spills into the photoreceptor plasma membrane domain (Figure 1A). This pattern is consistent with the expression of other membrane proteins that lack specific targeting information [30] and is most likely explained by the majority of the construct being non-specifically packaged into rhodopsin carrier vesicles, which are thought to comprise the majority of all transport vesicles in frog photoreceptors. The rest of the construct is likely to be non-specifically packaged into transport vesicles carrying membrane proteins to other subcellular compartments. When the YFP‐xRhoCTΔ5 reporter is fused with a specific outer segment targeting motif, such as the VXPX motif of rhodopsin [7] or the C-terminal domain of peripherin [15], it is localized exclusively to the outer segment. Therefore, specificity of outer segment targeting in this approach is indicated by the lack of the construct’s “spillage” outside the outer segment [7], [15], [30].

Previous study of peripherin’s C-terminus found that deletions affecting a twenty amino acid stretch (residues 317–336; we will refer to *Xenopus* peripherin sequence as xPer) resulted in loss of exclusive targeting to outer segments [15]. However, it was not examined if this sequence alone was sufficient to target a reporter to the outer segment. Therefore, we first investigated whether peripherin’s reported targeting sequence is able to specifically direct the YFP‐xRhoCTΔ5 reporter to the outer segment and found that it was (Figure 1B). We next asked whether all twenty amino acids are required for targeting, particularly because the first ten amino acids in this sequence overlap with peripherin’s previously identified fusogenic region located within residues 311–325 [24], [27]. In order to test whether these two functional regions overlap or are separate from one another, we generated constructs in which the reporter was fused to either the first ten amino acids or the last ten amino acids of the targeting region (Figure 1C, D). Only the latter construct targeted exclusively to the outer segment demonstrating that the peripherin targeting signal is wholly contained within a ten amino acid residue stretch and that it is distinct from peripherin’s fusogenic region.

Interestingly, the expression of the construct containing a portion of peripherin’s fusogenic region caused outer segments to appear distorted, which is particularly well-seen in rods displaying strong fluorescent signal (Figure 1C). Our construct (YFP‐xRhoCTΔ5-xPer 317–326) encompasses a part of an amino acid sequence promoting membrane fusion *in vitro*, including two of the three residues most critical for this function, Glu321 and Lys324 [24], [31]. This may explain why expression of this construct disrupts outer segment membranes. However, expression of a longer C-terminal construct did not result in irregular outer segment morphology (Figure 1A). One potential explanation for this difference is that membrane fusion by peripherin is likely to be a highly regulated process that occurs only during disc morphogenesis. Accordingly, this process would need to be prevented during the rest of the lifetime of peripherin. It could be further speculated that inhibitors of peripherin’s fusogenic activity can interact with longer, but not shorter, transgenic constructs thereby preventing disruption of outer segment membranes.

### Peripherin Targeting is Dependent on a Critical Valine Residue

**Figure 2:**
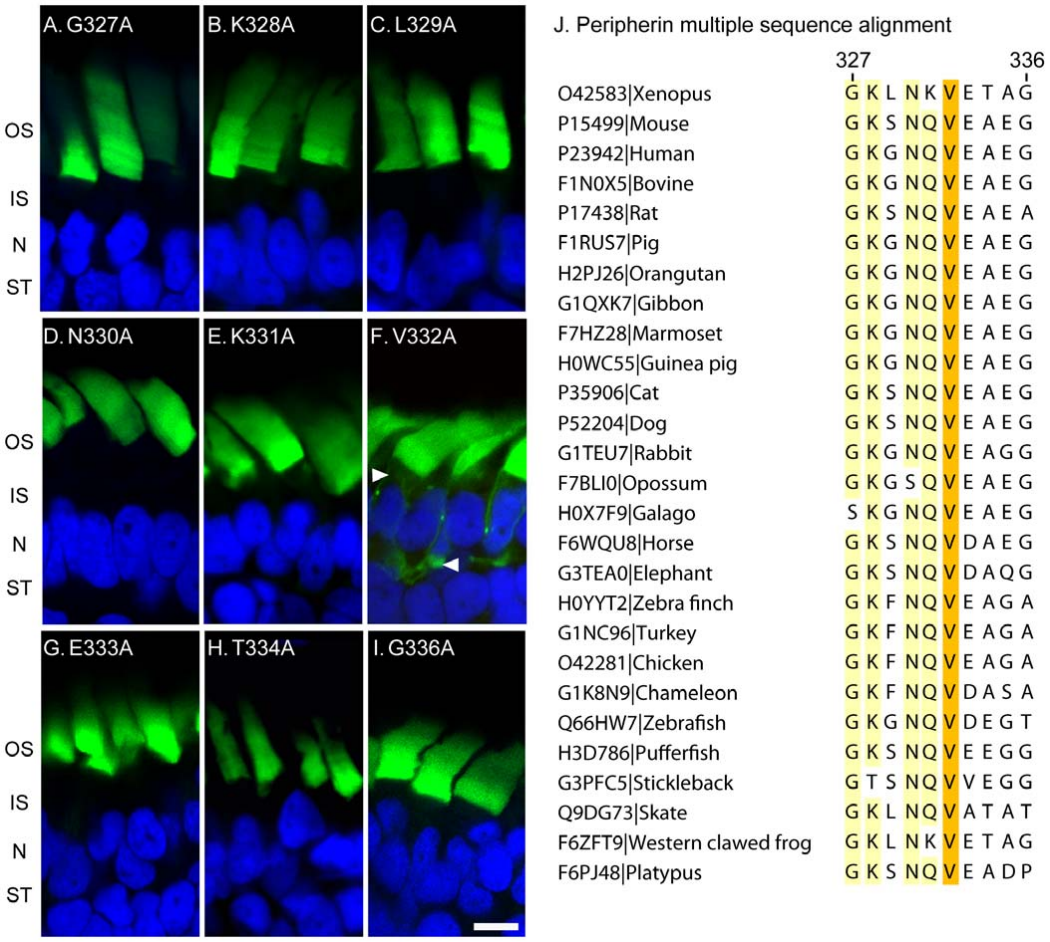
Alanine scanning mutagenesis of the peripherin targeting sequence identifies V332 as the only residue essential for targeting. Panels A–I show confocal images of transgenic frog retinas expressing the YFP-xRhoCTΔ5-xPer317–336 (green) variations containing single alanine substitutions of individual residues. (J) Multiple sequence alignment of peripherin targeting sequences from different species; residues with over 90% identity are highlighted yellow and the 100% conserved valine is highlighted orange. Abbreviations are: OS – outer segment, IS – inner segment, N – nuclei, ST – synaptic termini. Nuclei (blue) are stained with Hoechst. Scale bar 5 µm.

In the next set of experiments we tested which residues from peripherin’s 327–336 sequence are critical for outer segment targeting. We generated three peripherin reporter constructs containing overlapping 5-alanine substitutions of sequential amino acids within this sequence and found that none of these constructs were able to target exclusively to the outer segment (Figure 1E–G). This suggested that multiple residues within peripherin’s targeting sequence may be critical, which prompted us to mutagenize each one individually (Figure 2). Surprisingly, we discovered that only one residue, V332, was essential for proper reporter targeting (Figure 2F). All other mutants were faithfully delivered to the outer segment.

Examination of the corresponding sequence in peripherins from other species indicates that this valine is absolutely conserved in all species (Figure 2J), consistent with our experimental evidence of its functional importance. Notably, in a preceding experiment (Figure 1E), one of the constructs had five amino acids replaced with alanines upstream from an intact V332. This construct was mistargeted, demonstrating that while the V332 residue is essential, it is not a sole determinant for peripherin targeting.

### Peripherin Targeting Sequence can Redirect Subcellular Localization of Other Proteins

An alternative approach to characterize the sufficiency of peripherin’s targeting sequence for outer segment protein delivery is to test whether it could redirect intracellular trafficking of a protein reporter otherwise targeted to another subcellular compartment. For this purpose, we selected the Htr1a serotonin receptor because it was previously shown to be excluded from cilia in other cell types [32]. On the other hand, when fused to the rhodopsin C-terminus (including the VXPX signal) this receptor was shown to be delivered to rod outer segments of transgenic *Xenopus* [33].

**Figure 3:**
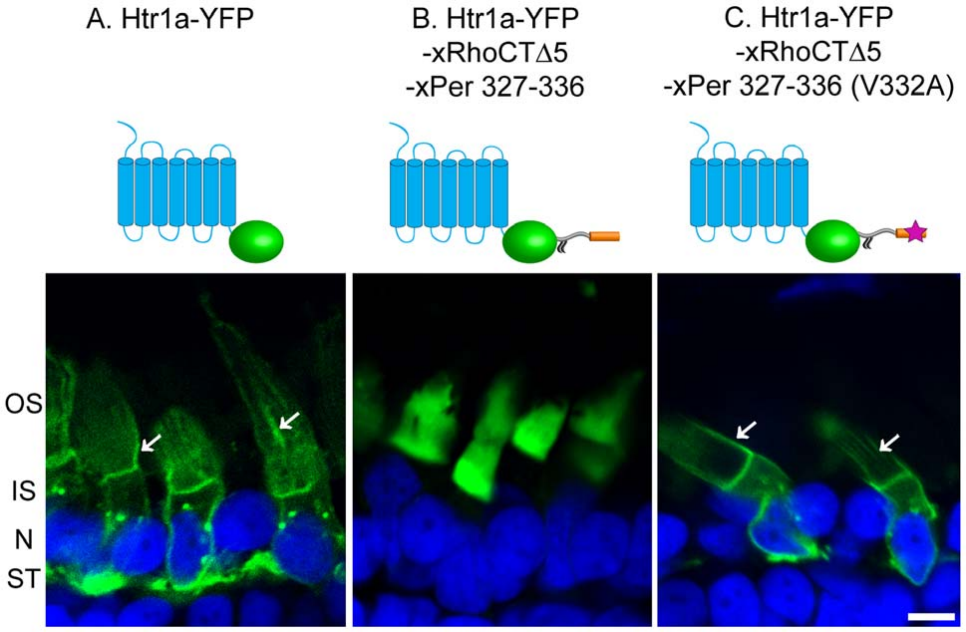
The peripherin targeting sequence redirects the subcellular targeting of the Htr1a serotonin receptor. Panels show confocal images of transgenic frog retinas expressing YFP-fused Htr1a constructs (green) schematically illustrated above each panel. (A) Htr1a-YFP; fluorescent signal observed in calycal processes is marked by white arrows. (B) Htr1a-YFP containing the Per327–336 sequence fused at the C-terminus. (C) Same construct as in (B), but bearing the V332A mutation. Abbreviations are: OS – outer segment, IS – inner segment, N – nuclei, ST – synaptic termini. Nuclei (blue) are stained with Hoechst. Scale bar: 5 µm.

We first demonstrated that the YFP-fused Htr1a construct was completely excluded from rod outer segments of transgenic *Xenopus*, in agreement with observations in other ciliated cells (Figure 3A). This construct distributed throughout the plasma membrane of the inner segment and synaptic terminal and was prominently present in the calycal processes, which are microvillar extensions of the inner segment plasma membrane surrounding the outer segment ([34] white arrowheads in Figures 3A and 3C). We next expressed the Htr1a-YFP construct fused to peripherin’s ten amino acid targeting sequence and found that it was localized exclusively to the outer segment (Figure 3B). This result shows that the peripherin targeting signal is sorted in such a way that it overrides all other targeting information contained within Htr1a and redirects it to the outer segment, just as the rhodopsin targeting sequence did [33]. We next demonstrated that Htr1a re-targeting was completely abrogated when the critical valine residue within peripherin’s targeting sequence was substituted for an alanine (Figure 3C). This result confirms the critical role of V332 and conclusively demonstrates that the absence of spillage seen with the original reporter fused to peripherin’s targeting sequence was not a result of its very efficient degradation in the inner segment.

### Outer Segment Targeting of Full-length Peripherin is Dependent on V332

Our next task was to demonstrate that V332 is critical for targeting full-length peripherin. The challenge of these experiments was the property of peripherin to form high order oligomers [35]–[37]. Consequently, any exogenously expressed peripherin mutant may oligomerize with endogenous peripherin, making it difficult to distinguish whether its intracellular distribution is determined by its own targeting information or information contained within higher order oligomers. For example, C-terminally truncated peripherin was targeted to outer segments of transgenic *Xenopus*, presumably due to oligomerization with the endogenous protein [22]. Therefore, we switched from frogs to mice, taking advantage of the *rds* mouse model lacking endogenous peripherin. Photoreceptors of these mice do not form outer segments [18], a phenotype restored upon transgenic peripherin expression [38]–[41].

**Figure 4:**
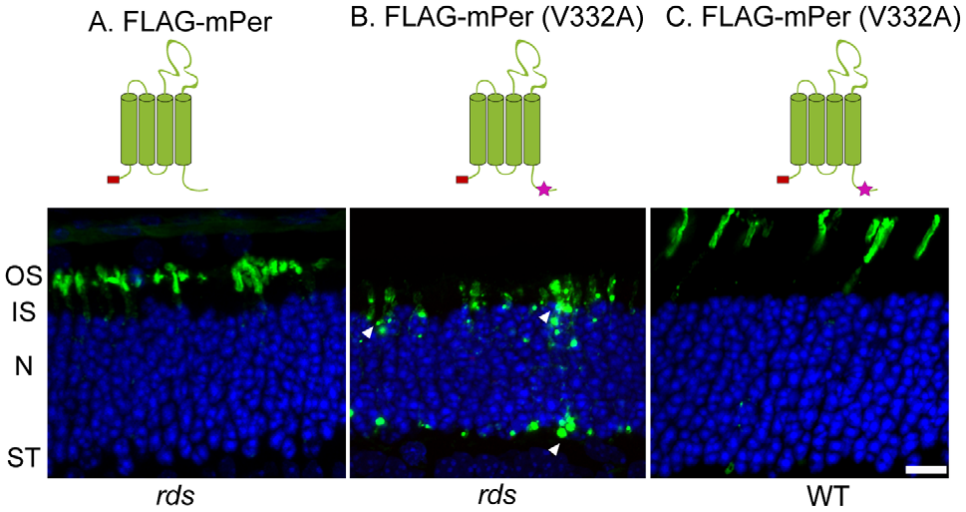
The V332A mutation abrogates peripherin’s outer segment targeting in rods of the *rds* mouse. (A) Wild type FLAG-tagged mouse peripherin (mPer) was electroporated into rods of the *rds* mouse and stained with anti-FLAG antibodies (green). (B) V332A FLAG-tagged peripherin electroporated into rods of the *rds* mouse; construct mislocalization to the inner segment and synaptic terminal is highlighted by white arrowheads. (C) V332A FLAG-tagged peripherin electroporated into the rods of wild type mice. The electroporated constructs are illustrated schematically above the corresponding panels with the position of the FLAG tag depicted in red. Note that the outer segment shapes in (A) and (C) are different due to ongoing photoreceptor degeneration in the *rds* mouse (A). Abbreviations are: OS – outer segment, IS – inner segment, N – nuclei, ST – synaptic termini. Nuclei (blue) are stained with Hoechst. Scale bar: 10 µm.

We expressed FLAG-tagged full-length wild type peripherin or its V332A mutant in rods of *rds* mice under control of the rhodopsin promoter. As shown in Figure 4A, the wild type peripherin construct restored the formation of rod outer segments in these mice and was localized nearly exclusively to this compartment. However, the V332A mutant was aggregated throughout expressing rods and did not promote outer segment formation (Figure 4B). In the final, control experiment, we expressed the V332A mutant in rods of wild type mice and found that it localized to the outer segment (Figure 4C; as found in a previous study with transgenic *Xenopus* [22]). This demonstrates that the V332A mutation does not affect peripherin’s ability to traffic to outer segments when oligomerization with endogenous wild type peripherin is allowed.

### Concluding Remarks

Our experiments demonstrate that a short C-terminal sequence is sufficient for outer segment targeting of peripherin. This sequence does not overlap with other known functional regions of this protein and only one amino acid, V332, within this sequence is indispensable for outer segment targeting. Peripherin’s targeting sequence is unique and does not have notable homology with other proteins residing in the outer segment, although it is hard to overlook that both peripherin and rhodopsin contain a valine residue critical for their targeting. The difference is that rhodopsin targeting also relies on a second indispensable residue, a proline within the VXPX sequence. The significance of both proteins containing a critical valine is currently unclear and awaits further studies of accessory proteins sorting peripherin into post-Golgi transport vesicles headed to the outer segment. The same studies would ultimately reveal whether the unique targeting sequence of peripherin directs it into a distinct outer segment trafficking pathway, or if it merely directs peripherin into a common trafficking pathway with rhodopsin.

Genetic studies have not yet identified mutations within the targeting region of human peripherin to be associated with retinitis pigmentosa or similar retinal degenerations. However, our data in Figure 4C suggest that, unlike mutations affecting peripherin oligomerization, mutations affecting peripherin targeting would need to be homozygous in order to cause a disease phenotype, which would make most potential carriers non-phenotypic. On the other hand, individuals heterozygous for mutations causing peripherin truncations at positions 290 and 331(upstream from or in the middle of the targeting sequence) suffer from a condition called pattern dystrophy [42]. This condition is characterized by the deposition of abnormal pigment in the retinal pigment epithelium and may be associated with blurred vision and distortion of straight lines upon ophthalmic examination. Therefore, the complete pathobiological picture of mutations affecting peripherin targeting may be subtle and require a more detailed analysis in the future.

## Materials and Methods

### Animals

Frogs and mice were handled following the protocols (registry number A212-12-08 and A057-10-03, respectively) approved by the Institutional Animal Care and Use Committees of Duke University. Adult *Xenopus laevis* frogs were purchased from Nasco. Wild type CD1 mice were obtained from Charles River; *rds* mice were obtained from Jackson Laboratories. Frogs and mice were housed under a 12/12 hour diurnal cycle. Prior to egg laying, female adult frogs were housed in a multi-tank flow-through system under standard environmental conditions. Newly hatched tadpoles were maintained in large Petri dishes at a density of one animal per two ml.

### DNA Constructs

DNA constructs were generated using standard PCR-based subcloning methods. To generate all *Xenopus* YFP fusion constructs and their point mutants, overhang extension PCR primers were used (primer sequences available upon request). The FLAG tag was appended to full length mouse peripherin’s N-terminus by introducing the sequence into the forward primer. To make the V332A point mutation in mouse peripherin, we utilized the QuikChange II XL kit (Stratagene). All forward and reverse primers were designed to introduce 5′-AgeI and 3′-NotI sites respectively. PCR products were subcloned into either the XOP5.5 vector [43] for *Xenopus* transgenesis or pRho2.2 [44] for *in vivo* electroportation. DNA templates were obtained as follows: IMAGE clones corresponding to *Xenopus* peripherin (xRDS38), *Xenopus* rhodopsin, and mouse peripherin were purchased from American Type Culture Collection; YFP was cloned from pDV-NTAP-NYFP obtained from the The Dicty Stock Center; HTR1A was obtained from a mouse brain cDNA library (Stratagene). All constructs were confirmed by direct sequencing.

### Production of Transgenic Tadpoles

Transgenic *Xenopus* tadpoles were generated using the restriction enzyme-mediated integration method [45], [46] with modifications described in our previous studies [30], [47]. In brief, linearized plasmid DNA containing the transgene was mixed with *Xenopus* sperm nuclei in the presence of the restriction enzyme *XhoI* to promote integration. Transgenic sperm nuclei were then injected into *Xenopus* oocytes, and the resulting embryos were allowed to develop until stage 45–54 when tadpoles were sacrificed by immersion in 0.2% tricaine. A minimum of four positive transgenic animals were analyzed for each DNA construct.

### *In vivo* Electroporation of Mouse Retinas

Retinal transfection of neonatal mice was performed as described by Matsuda and Cepko [44]. Following anesthetization of neonatal mice on ice, the eyelid and sclera were punctured at the periphery of the eye using a 30 gauge needle. A blunt-end 32 gauge needle was advanced through the puncture wound until reaching the subretinal space, and 0.3–0.5 µl of concentrated plasmid DNA (4 µg/µl of the construct of interest and 2 µg/µl DsRed) was deposited. A tweezer-type electrode (BTX) was placed over the mouse’s head with the positive electrode overlying the injected eye. Five 100–110 V pulses of 50 ms duration were applied using an ECM830 square pulse generator (BTX). Neonates were returned to their mother and allowed to develop until postnatal day 21 when mice were sacrificed by CO_2_ inhalation followed by decapitation. A minimum of three positively expressing mice retinas were analyzed for each DNA construct.

### Immunofluorescence

Posterior eyecups from mouse eyes were obtained by microdissection, fixed in 4% paraformaldehyde for 1 hour, rinsed three times in PBS, and embedded in 4.0% agarose (Invitrogen). A vibratome (Leica VT1200S) was used to collect 100****µm cross-sections through the central retina. Sections were collected in 24-well plates, blocked in 5% goat serum in the presence of 0.5% Triton X-100, incubated overnight with monoclonal mouse anti-FLAG M2 (Sigma-Aldrich) diluted in blocker, and stained with goat anti-mouse Alexa Fluor 488 secondary antibody (Invitrogen) and 10 µg/ml Hoechst 33342 (Invitrogen) to label nuclei. To examine the eyes of transgenic tadpoles, the entire animal was fixed in 4% paraformaldehyde, cryoprotected in 30% sucrose, and frozen in 100% tissue-freezing medium. 12****µm cross-sections through the eye were collected directly onto glass slides and stained with 2 µg/ml Hoechst to label the nuclei.

All samples were mounted with Fluoromount (Electron Microscopy Sciences) under glass coverslips and visualized using a Nikon Eclipse 90i microscope and a C1 confocal scanner controlled by EZ-C1 v 3.10 software (Nikon). Manipulation of images was limited to adjusting the brightness level, image size, and cropping using either EZ-C1 v 3.10 Viewer or Photoshop (Adobe).

## References

[1] Berger W, Kloeckener-Gruissem B, Neidhardt J (2010) The molecular basis of human retinal and vitreoretinal diseases. Prog Retin Eye Res 29: 335–375. 20362068 10.1016/j.preteyeres.2010.03.004

[2] Bessant DA, Khaliq S, Hameed A, Anwar K, Payne AM, et al (1999) Severe autosomal dominant retinitis pigmentosa caused by a novel rhodopsin mutation (Ter349Glu). Mutations in brief no. 208. Online. Hum Mutat 13: 83. 10.1002/(SICI)1098-1004(1999)13:1<83::AID-HUMU12>3.0.CO;2-5 10189219

[3] Berson EL, Rosner B, Weigel-DiFranco C, Dryja TP, Sandberg MA (2002) Disease progression in patients with dominant retinitis pigmentosa and rhodopsin mutations. Invest Ophthalmol Vis Sci 43: 3027–3036. 12202526

[4] De Matteis MA, Luini A (2008) Exiting the Golgi complex. Nat Rev Mol Cell Biol 9: 273–284. 18354421 10.1038/nrm2378

[5] Rodriguez-Boulan E, Kreitzer G, Musch A (2005) Organization of vesicular trafficking in epithelia. Nat Rev Mol Cell Biol 6: 233–247. 15738988 10.1038/nrm1593

[6] Deretic D, Schmerl S, Hargrave PA, Arendt A, McDowell JH (1998) Regulation of sorting and post-Golgi trafficking of rhodopsin by its C-terminal sequence QVS(A)PA. Proc Natl Acad Sci USA 95: 10620–10625. 9724753 10.1073/pnas.95.18.10620 PMC27944

[7] Tam BM, Moritz OL, Hurd LB, Papermaster DS (2000) Identification of an outer segment targeting signal in the COOH terminus of rhodopsin using transgenic *Xenopus laevis* . J Cell Biol 151: 1369–1380. 11134067 10.1083/jcb.151.7.1369 PMC2150681

[8] Luo W, Marsh-Armstrong N, Rattner A, Nathans J (2004) An outer segment localization signal at the C terminus of the photoreceptor-specific retinol dehydrogenase. J Neurosci 24: 2623–2632. 15028754 10.1523/JNEUROSCI.5302-03.2004 PMC6729528

[9] Sung CH, Makino C, Baylor D, Nathans J (1994) A Rhodopsin Gene Mutation Responsible for Autosomal-Dominant Retinitis-Pigmentosa Results in a Protein That Is Defective in Localization to the Photoreceptor Outer Segment. J Neurosci 14: 5818–5833. 7523628 10.1523/JNEUROSCI.14-10-05818.1994 PMC6576989

[10] Geng L, Okuhara D, Yu ZH, Tian X, Cai YQ, et al (2006) Polycystin-2 traffics to cilia independently of polycystin-1 by using an N-terminal RVxP motif. J Cell Sci 119: 1383–1395. 16537653 10.1242/jcs.02818

[11] Ward HH, Brown-Glaberman U, Wang J, Morita Y, Alper SL, et al (2011) A conserved signal and GTPase complex are required for the ciliary transport of polycystin-1. Mol Biol Cell 22: 3289–3305. 21775626 10.1091/mbc.E11-01-0082 PMC3172256

[12] Jenkins PM, Hurd TW, Zhang L, McEwen DP, Brown RL, et al (2006) Ciliary targeting of olfactory CNG channels requires the CNGB1b subunit and the kinesin-2 motor protein, KIF17. Curr Biol 16: 1211–1216. 16782012 10.1016/j.cub.2006.04.034

[13] Deretic D, Williams AH, Ransom N, Morel V, Hargrave PA, et al (2005) Rhodopsin C terminus, the site of mutations causing retinal disease, regulates trafficking by binding to ADP-ribosylation factor 4 (ARF4). Proc Natl Acad Sci USA 102: 3301–3306. 15728366 10.1073/pnas.0500095102 PMC552909

[14] Deretic D, Wang J (2012) Molecular assemblies that control rhodopsin transport to the cilia. Vision Res. doi: http://dx.doi.org/10.1016/j.visres.2012.07.015. 10.1016/j.visres.2012.07.015 PMC3514645 22892112

[15] Tam BM, Moritz OL, Papermaster DS (2004) The C terminus of peripherin/rds participates in rod outer segment targeting and alignment of disk incisures. Mol Biol Cell 15: 2027–2037. 14767063 10.1091/mbc.E03-09-0650 PMC379296

[16] Molday LL, Arikawa K, Illing M, Williams DS, Molday RS (1992) Localization and Molecular Characterization of Peripherin Rds in Outer Segments of Rod and Cone Photoreceptor Cells. Invest Ophthalmol Vis Sci 33: 740–740.

[17] Arikawa K, Molday LL, Molday RS, Williams DS (1992) Localization of Peripherin/Rds in the Disk Membranes of Cone and Rod Photoreceptors - Relationship to Disk Membrane Morphogenesis and Retinal Degeneration. J Cell Biol 116: 659–667. 1730772 10.1083/jcb.116.3.659 PMC2289304

[18] Travis GH, Brennan MB, Danielson PE, Kozak CA, Sutcliffe JG (1989) Identification of a photoreceptor-specific mRNA encoded by the gene responsible for retinal degeneration slow (rds). Nature 338: 70–73. 2918924 10.1038/338070a0

[19] Sanyal S, De Ruiter A, Hawkins RK (1980) Development and degeneration of retina in rds mutant mice: light microscopy. J Comp Neurol 194: 193–207. 7440795 10.1002/cne.901940110

[20] Sanyal S, Jansen HG (1981) Absence of receptor outer segments in the retina of rds mutant mice. Neurosci Lett 21: 23–26. 7207866 10.1016/0304-3940(81)90051-3

[21] Fariss RN, Matsumoto B, Molday RS, Fisher SK (1992) Induced Photoreceptor Degeneration Leads to Changes in the Immunolocalization of the Photoreceptor Proteins Opsin and Peripherin/Rds. Mol Biol Cell 3: A56–A56.

[22] Lee ES, Burnside B, Flannery JG (2006) Characterization of peripherin/rds and rom-1 transport in rod photoreceptors of transgenic and knockout animals. Invest Ophthalmol Vis Sci 47: 2150–2160. 16639027 10.1167/iovs.05-0919 PMC1950294

[23] Hagstrom SA, Duyao M, North MA, Li TS (1999) Retinal degeneration in tulp1^−/−^ mice: Vesicular accumulation in the interphotoreceptor matrix. Invest Ophthalmol Vis Sci 40: 2795–2802. 10549638

[24] Boesze-Battaglia K, Goldberg AFX, Dispoto J, Katragadda M, Cesarone G, et al (2003) A soluble peripherin/Rds C-terminal polypeptide promotes membrane fusion and changes conformation upon membrane association. Exp Eye Res 77: 505–514. 12957149 10.1016/s0014-4835(03)00151-9 PMC4732724

[25] Edrington TC, Lapointe R, Yeagle PL, Gretzula CL, Boesze-Battaglia K (2007) Peripherin-2: An intracellular analogy to viral fusion proteins. Biochemistry 46: 3605–3613. 17323921 10.1021/bi061820c

[26] Boesze-Battaglia K, Goldberg AFX (2002) Photoreceptor renewal: A role for peripherin/rds. Int Rev Cell Mol Biol, Vol 217 217: 183–225. 10.1016/s0074-7696(02)17015-x PMC4732730 12019563

[27] Boesze-Battaglia K, Lamba OP, Napoli AA, Sinha S, Guo YQ (1998) Fusion between-retinal rod outer segment membranes and model membranes: A role for photoreceptor peripherin/rds. Biochemistry 37: 9477–9487. 9649331 10.1021/bi980173p

[28] Bonifacino JS, Traub LM (2003) Signals for sorting of transmembrane proteins to endosomes and lysosomes. Annu Rev Biochem 72: 395–447. 12651740 10.1146/annurev.biochem.72.121801.161800

[29] Pandey KN (2010) Small peptide recognition sequence for intracellular sorting. Curr Opin Biotechnol 21: 611–620. 20817434 10.1016/j.copbio.2010.08.007 PMC2997389

[30] Baker SA, Haeri M, Yoo P, Gospe SM, Skiba NP, et al (2008) The outer segment serves as a default destination for the trafficking of membrane proteins in photoreceptors. J Cell Biol 183: 485–498. 18981232 10.1083/jcb.200806009 PMC2575789

[31] Ritter LM, Boesze-Battaglia K, Tam BM, Moritz OL, Khattree N, et al (2004) Uncoupling of photoreceptor peripherin/rds fusogenic activity from biosynthesis, subunit assembly, and targeting - A potential mechanism for pathogenic effects. J Biol Chem 279: 39958–39967. 15252042 10.1074/jbc.M403943200 PMC1360210

[32] Berbari NF, Johnson AD, Lewis JS, Askwith CC, Mykytyn K (2008) Identification of ciliary localization sequences within the third intracellular loop of G protein-coupled receptors. Mol Biol Cell 19: 1540–1547. 18256283 10.1091/mbc.E07-09-0942 PMC2291422

[33] Zhang L, Salom D, He J, Okun A, Ballesteros J, et al (2005) Expression of functional G protein-coupled receptors in photoreceptors of transgenic *Xenopus laevis* . Biochemistry 44: 14509–14518. 16262251 10.1021/bi051386z

[34] Peters KR, Palade GE, Schneider BG, Papermaster DS (1983) Fine structure of a periciliary ridge complex of frog retinal rod cells revealed by ultrahigh resolution scanning electron microscopy. J Cell Biol 96: 265–276. 6219117 10.1083/jcb.96.1.265 PMC2112274

[35] Loewen CJR, Molday RS (2000) Disulfide-mediated oligomerization of peripherin/Rds and Rom-1 in photoreceptor disk membranes - Implications for photoreceptor outer segment morphogenesis and degeneration. J Biol Chem 275: 5370–5378. 10681511 10.1074/jbc.275.8.5370

[36] Goldberg AFX, Moritz OL, Molday RS (1995) Heterologous Expression of Photoreceptor Peripherin/Rds and Rom-1 in Cos-1 Cells - Assembly, Interactions, and Localization of Multisubunit Complexes. Biochemistry 34: 14213–14219. 7578020 10.1021/bi00043a028

[37] Goldberg AFX, Molday RS (1996) Subunit composition of the peripherin/rds-rom-1 disk rim complex from rod photoreceptors: Hydrodynamic evidence for a tetrameric quaternary structure. Biochemistry 35: 6144–6149. 8634257 10.1021/bi960259n

[38] Nour M, Fliesler SJ, Naash MI (2008) Genetic supplementation of RDS alleviates a loss-of-function phenotype in C214S model of retinitis pigmentosa. Adv Exp Med Biol 613: 129–138. 18188937 10.1007/978-0-387-74904-4_14 PMC2789457

[39] Travis GH, Groshan KR, Lloyd M, Bok D (1992) Complete rescue of photoreceptor dysplasia and degeneration in transgenic retinal degeneration slow (rds) mice. Neuron 9: 113–119. 1385966 10.1016/0896-6273(92)90226-4

[40] Ali RR, Sarra GM, Stephens C, Alwis MD, Bainbridge JW, et al (2000) Restoration of photoreceptor ultrastructure and function in retinal degeneration slow mice by gene therapy. Nat Genet 25: 306–310. 10888879 10.1038/77068

[41] Nour M, Naash MI, Fliesler SJ (2004) Supplementation of peripherin/rds rescues mutation-associated rod and cone photoreceptor defects in transgenic mice. Mol Ther 9: S89–S89.

[42] Grover S, Fishman GA, Stone EM (2002) Atypical presentation of pattern dystrophy in two families with peripherin/RDS mutations. Ophthalmology 109: 1110–1117. 12045052 10.1016/s0161-6420(02)01029-1

[43] Knox BE, Schlueter C, Sanger BM, Green CB, Besharse JC (1998) Transgene expression in Xenopus rods. FEBS Lett 423: 117–121. 9512341 10.1016/s0014-5793(98)00018-0

[44] Matsuda T, Cepko CL (2004) Electroporation and RNA interference in the rodent retina in vivo and in vitro. Proc Natl Acad Sci USA 101: 16–22. 14603031 10.1073/pnas.2235688100 PMC314130

[45] Amaya E, Kroll KL (1999) A method for generating transgenic frog embryos. Methods Mol Biol 97: 393–414. 10443381 10.1385/1-59259-270-8:393

[46] Kroll KL, Amaya E (1996) Transgenic Xenopus embryos from sperm nuclear transplantations reveal FGF signaling requirements during gastrulation. Development 122: 3173–3183. 8898230 10.1242/dev.122.10.3173

[47] Gospe SM, Baker SA, Arshavsky VY (2010) Facilitative glucose transporter Glut1 is actively excluded from rod outer segments. J Cell Sci 123: 3639–3644. 20923839 10.1242/jcs.072389 PMC2964109

